# The application of CGF combined with GBR in alveolar bone increment for patients with anxiety disorder: A rare case report and literature review

**DOI:** 10.1097/MD.0000000000035905

**Published:** 2023-11-10

**Authors:** Zhixin Li, Chao Yang, Jinrong Wang, Kaiyue Zheng, Wen Luo

**Affiliations:** a Department of Stomatology, First Affiliated Hospital of Hainan Medical University, Haikou, China; b School of Stomatology, Hainan Medical University, Haikou, China; c Department of Stomatology, The People’s Hospital of Longhua, Shenzhen, China; d Research and Development Department, Shenzhen Uni-medica Technology CO., Ltd, Shenzhen, China.

**Keywords:** anxiety disorder, implant, concentrated growth factor, guided bone regeneration, alveolar bone increment

## Abstract

**Rationale:**

Selective serotonin reuptake inhibitors (SSRIs), one of the commonly used anti-anxiety drugs, may have impacts on bone metabolism and potentially lead to drug-induced osteoporosis. The traditional approach of oral implantation in individuals with both anxiety disorder and drug-induced osteoporosis poses a significant challenge. To address this issue, concentrated growth factor (CGF) has been utilized in patients undergoing concurrent alveolar ridge augmentation during oral implantation, resulting in favorable clinical outcomes. Consequently, combining CGF with guided bone regeneration (GBR) in alveolar bone increment may represent a promising new surgical approach for such patients. In this report, we present a case study of a 25-year-old male with anxiety disorder and drug-induced osteoporosis, in who CGF combined with GBR was employed in alveolar bone increment.

**Patient concerns:**

This article reports the case of a 25-year-old male who underwent cone beam computed tomography (CBCT) due to the absence of his right lower second molar for a period of six months. The CBCT scan revealed significant bone defects, which were attributed to the tooth loss and prolonged use of anti-anxiety drugs. Consequently, the patient sought medical assistance from our department.

**Diagnoses:**

Based on the patient's self-report, he was diagnosed with an anxiety disorder. Additionally, the CBCT scan confirmed the loss of the right mandibular second molar and revealed the presence of dental irregularity and an alveolar bone defect.

**Interventions:**

During the patient's course of treatment with anti-anxiety medication, a combination of CGF and GBR was employed for the simultaneous implantation of the missing right mandibular second molar, along with bone augmentation.

**Outcomes:**

The patient had a follow-up visit two weeks after the surgical procedure, and the wound in the operation area had healed satisfactorily. Six months later, CBCT images revealed excellent osseointegration. The buccal and lingual width of the alveolar bone measured 6.95mm, which was an increase of 1.35mm compared to the pre-implantation stage.

**Lessons:**

This article presents a case study in which CGF combined with GBR were utilized to address alveolar bone augmentation during the implantation phase in patients taking anti-anxiety medication. The results demonstrated that CGF combined with GBR, as a cutting-edge platelet concentrate technique, could effectively stimulate bone tissue proliferation in individuals who have been on long-term anti-anxiety medication, specifically in oral implant areas. This approach can help prevent poor osseointegration, promote higher osseointegration rates, and facilitate wound healing.

## 1. Introduction

Anxiety disorder is widely described as a group of diseases that cause anxiety symptoms with unknown causes, The common clinical manifestations are anxiety and fear.^[1]^ Selective serotonin reuptake inhibitors (SSRIs) are one of the commonly used anti-anxiety drugs. Studies have shown that the concentration of such drugs in bone marrow is higher than that in brain or blood, which will increase bone fragility and fracture risk.^[2]^ In implant restoration, alveolar bone is an important anatomical basis for oral implant restoration. Adequate and good quality alveolar bone is the key to successful implantation and long-term stability of implants, and it is also an important factor in preventing disease around the implants.^[3,4]^ In clinical work, cases of insufficient bone mass in the edentulous area are often encountered,^[5,6]^ bone increment is often required before or implanting simultaneous to restore the deficiency of alveolar bone mass.^[7,8]^ The degree of bone defect determines the level of bone augmentation surgery. However, it’s final quality, quantity and shape reconstruction effect will also be affected by many factors in the treatment process. Objective guided bone regeneration (GBR) is a bone augmentation technique commonly used in clinical alveolar bone deficiency.^[9]^ In the course of treatment, but occasionally complications occur, such as bone resorption, bone cracking, etc. In previous study, concentrated growth factor (CGF) as the latest generation of platelet concentrate,^[10]^ can promote bone regeneration and reduce the incidence of postoperative complications for bone reconstruction. This article will report a patient with anxiety disorder and long-term use of related drugs in the mandibular second molar implant at the same time using CGF combined with GBR technology for alveolar ridge increment. By measuring the results of cone-beam computed tomography (CBCT) bone height and width before and after operation, the effect of CGF combined with GBR technology on alveolar bone increment in patients with anxiety disorder was discussed, and the relevant literature was reviewed in order to provide an effective reference for the clinical diagnosis and treatment of such special patients.

## 2. Clinical data

### 2.1. General information

The patient, male, 25 years old, was admitted to the Department of Prosthodontics of First Affiliated Hospital of Hainan Medical University in 2022 for more than half a year of missing right mandibular second molar and requiring repair. The patient was correcting in the Department of Orthodontics of First Affiliated Hospital of Hainan Medical University, he had communicated with the orthodontist and could first implant right mandibular second molar. He had a history of mild anxiety for 2 years. He took fluvoxamine maleate tablets (Lizhu Group Lizhu Pharmaceutical Factory, China), quetiapine fumarate tablets (Suzhou First Pharmaceutical Co, Ltd., China) and alprazolam tablets (Jiangsu Enhua Pharmaceutical Co. Ltd., China) for 2 years. He denied other systemic diseases and drug allergy history. Patients were informed and signed informed consent for implant surgery.

### 2.2. Dental examination

Traditional orthodontic brackets and square arch wires were visible in the upper and lower dentition; the right mandibular second molar missing, and there was no abnormality in the surrounding mucosa. The alveolar crest was narrow in the maxillofacial view. The buccal alveolar bone was absorbed in a slope shape, with obvious depression, less keratinized gingiva, shallow vestibular sulcus, and obvious elongation of the jaw teeth.

### 2.3. Auxiliary examination

**Figure 1.**
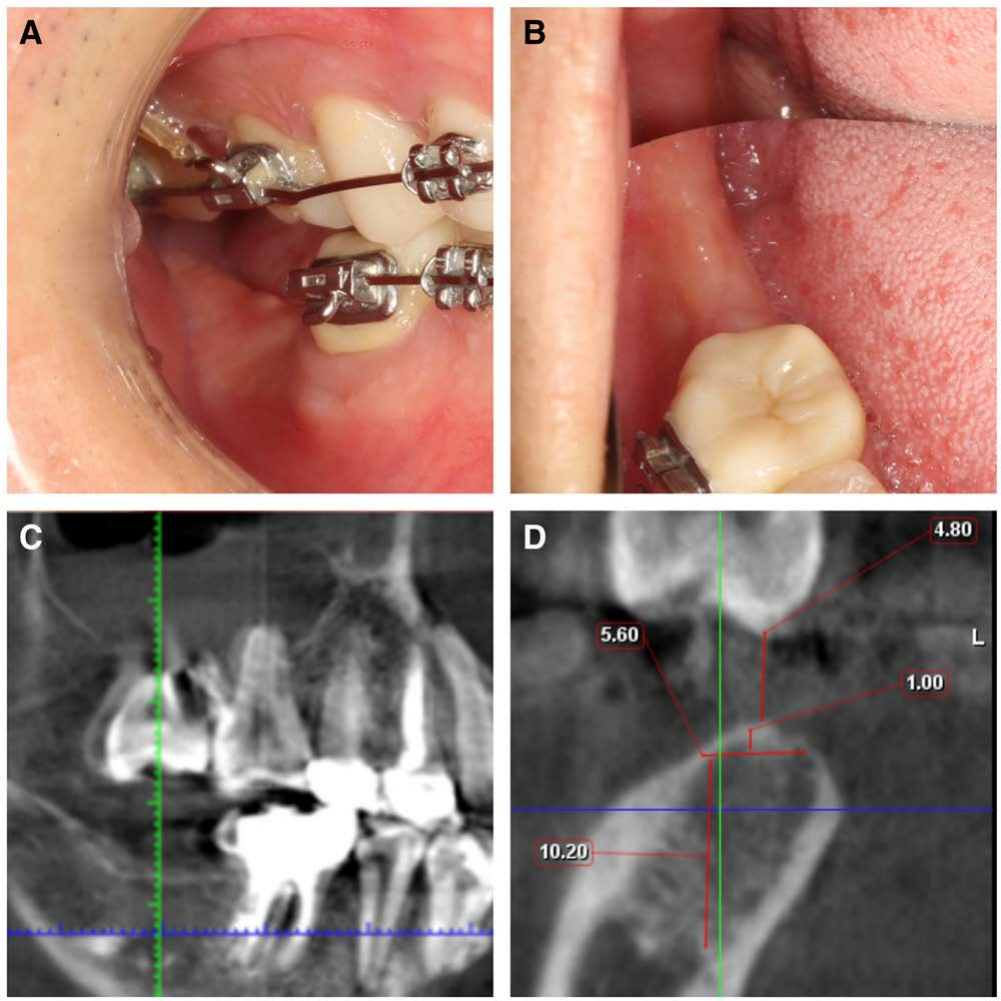
Preoperative assessment. (A) Right occlusion photo. (B) Occlusal photography. (C) Sagittal slice imaging of CBCT. (D) Coronal section screenshots. CBCT = cone-beam computed tomography.

CBCT results coronal section screenshots showed that the width of the buccal and lingual bone at 1 mm below the alveolar crest of 47 teeth was 5.6 mm, which was used as the vertical line. The distance from the mandibular canal was 10.2 mm, and the distance from the alveolar crest mucosa to the contralateral functional tip was 4.8 mm (Fig. 1).

### 2.4. Clinical diagnosis

Anxiety disorder; dental irregularity; and dentition defect.

### 2.5. Treatment plan

**Figure 2.**
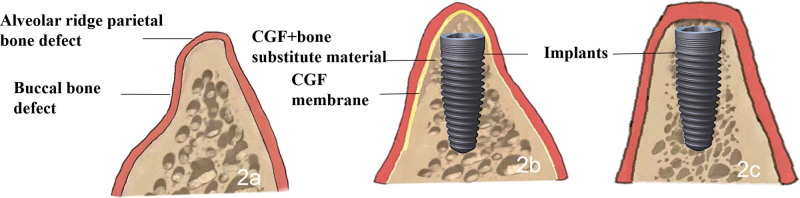
Schematic diagram of CGF combined with GBR. (A) Buccal and alveolar ridge parietal bone defects. (B) Bone defects were reconstructed with bone powder and CGF after implant implantation. (C) Postoperative after 6 mo. CGF = concentrated growth factor, GBR = guided bone regeneration.

Right mandibular second molar implants (ITI/BL 4.1 mm × 8 mm) were implanted, and CGF combined with GBR was performed for bone augmentation simultaneously (Fig. 2).

### 2.6. Treatment process

#### 2.6.1. Preoperative preparation.

Patients underwent routine oral examination, blood routine and coagulation function examination before operation to complete periodontal basic treatment. CBCT was taken before operation to evaluate the amount of bone defect and available bone mass. The patients were informed of the treatment process, cycle, cost, intraoperative and postoperative complications, and signed the informed consent of the implant surgery. The blood pressure was measured as 96/72 mm Hg, and cefuroxime axetil tablets (Zhuhai Federal Pharmaceutical Co., Ltd. China) were taken half an hour before the operation for routine anti-infection treatment.

#### 2.6.2. Preparation of CGF.

**Figure 3.**
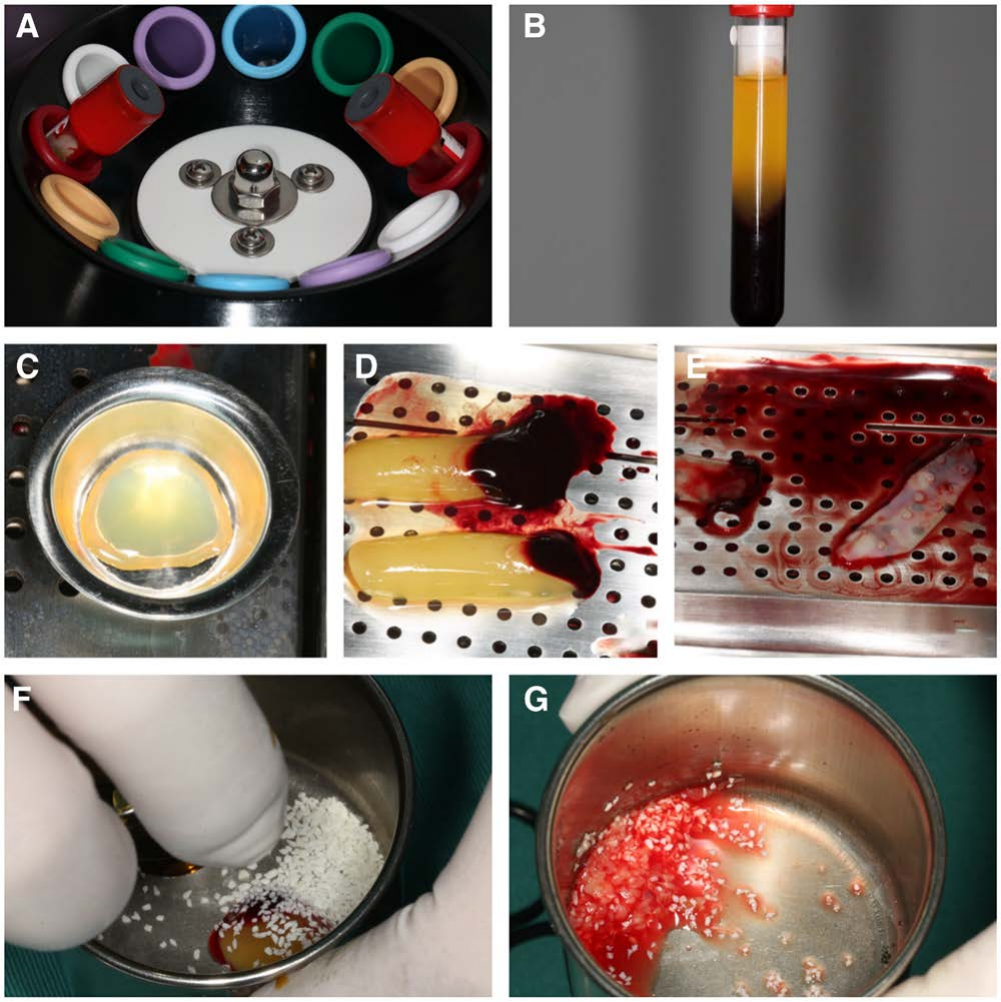
CGF production process. (A) Place the blood collection tube symmetrically in the corresponding position of the centrifuge. (B) CGF after centrifugation. (C) Collect the upper serum. (D) Place CGF on the film pressing button. (E) Pressed CGF film. (F) Mixing CGF with Bio-Gene bone Powder. (G) After mixing with the mixer. CGF = concentrated growth factor.

Two tubes of venous blood were collected with a 17-gauge needle and collected in two 10 mL glass-coated plastic tubes (Jiangsu Kangze Medical Device Co., Ltd., China) without anticoagulants. It was centrifuged using a centrifuge (Henan Duling Technology Co., Ltd., China) in the following manner: acceleration, 2400 to 2700 rpm Variable speed centrifugal system 12 minutes, until end. At the end of the procedure, 4 layers were obtained from bottom to top: red blood cell layer, growth factors and stem cell layer, buffy coating, serum layer platelet-poor plasma. Then, the CGF layer was separated using sterile surgical scissors. The prepared CGF fibrin gel was placed on a metal mesh plate and pressed into a CGF membrane. One of the CGF was cut into 1 to 2 mm particles with scissors, mixed with allogeneic bone repair material Bio-Gene 0.5 g (Beijing Daqing Biotechnology Co., Ltd., China) and mixed into a cup to paste (Fig. 3).

#### 2.6.3. Surgical procedure.

**Figure 4.**
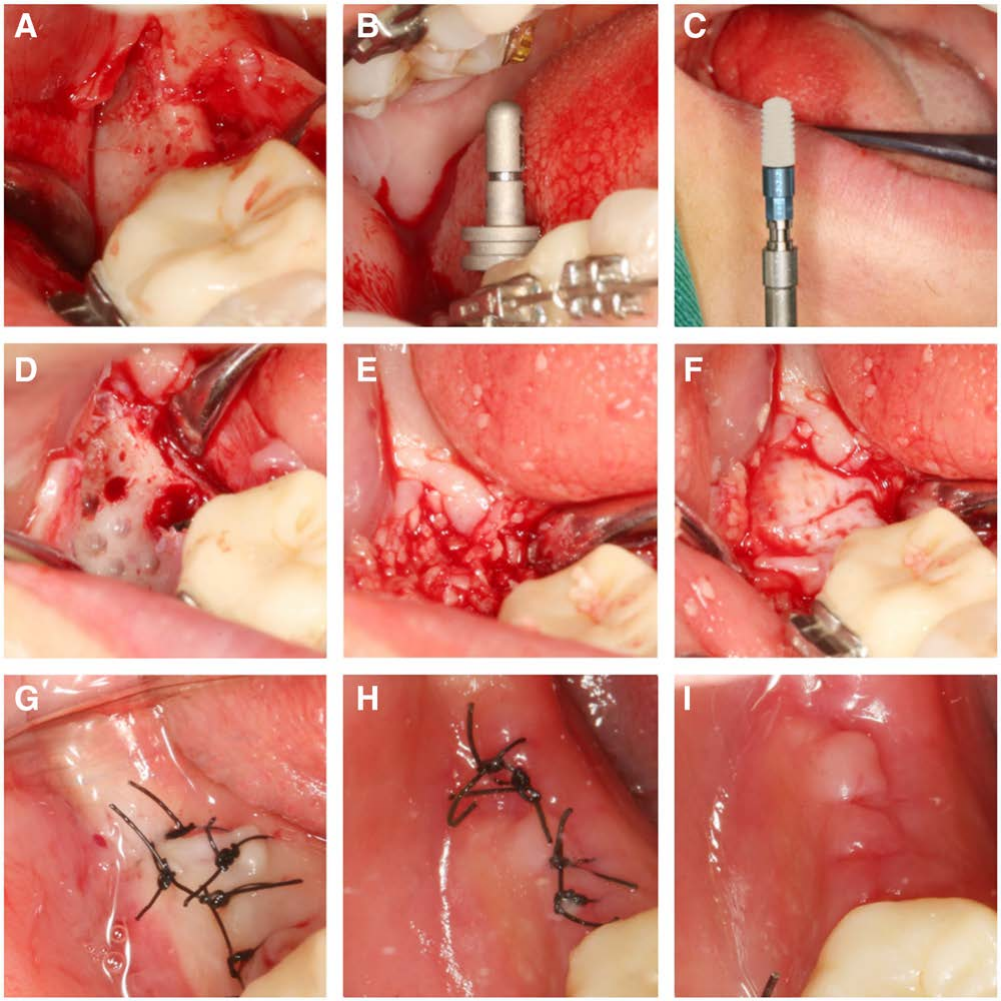
Surgical process. (A) Open the flap to expose the bone surface, and obvious bone resorption can be seen on the buccal side. (B) Parallel rod detection direction. (C) Implant implantation. (D) Preparation of buccal nutrient foramen. (E) Place CGF and Bio-Gene bone powder at the buccal and alveolar crest sites. (F) CGF membrane placed to cover the bone defect. (G) Tensionless suture. (H and I) Suture in place and wound healing recovered well. CGF = concentrated growth factor.

Patients with preoperative provide one iodine gargle (Chengdu Yong’an Pharmaceutical Co., Ltd., China) +0.9% saline solution (1:3) gargle 3 times, 1 minute each time. The maxillofacial region was disinfected with type III Anerdian disinfectant. The disinfection range was from the inferior orbital margin to the preauricular area on both sides of the clavicle. After local infiltration anesthesia with articaine epinephrine injection (1.7 mL, PIERRE ROLLAND, French), 47 alveolar crest incision was performed to reach the periosteum, and the incision was made in the gingival sulcus of the proximal adjacent teeth. An additional incision was made in the distal buccal side. The buccal and lingual mucoperiosteal flap was opened, the granulation tissue was removed, and the bone surface of 47 area was fully exposed. The alveolar crest and labial alveolar bone were partially missing. 47 site implant socket was prepared. The BL 4.1 × 8 mm implant was implanted, the torque was 15 Ncm, and the covering screw was installed. The buccal bone surface ball drill prepared the nourishing hole, and the drill passed through the cortical bone layer to increase the blood supply of the site. Buccal and lingual mucosal tension reduction was performed. The preoperatively prepared CGF and bone powder mixture was placed in the bone defect, and then 2 layers of CGF membrane were covered on the surface. The CGF membrane was extended to the palatal side of the alveolar ridge crest, and tension-free tight suture was performed using 4-0 non-absorbable suture. The patients were advised to take cephalosporin antibiotics, 3 times a day for 3 days, and 0.12% compound chlorhexidine gargle (Jiangsu Chenpai Bond Pharmaceutical Co., Ltd., China), 3 times a day for 1 week. The stitches were removed 14 days after surgery and the wound healing recovered well (Fig. 4).

#### 2.6.4. Secondary stage implant surgery.

**Figure 5.**
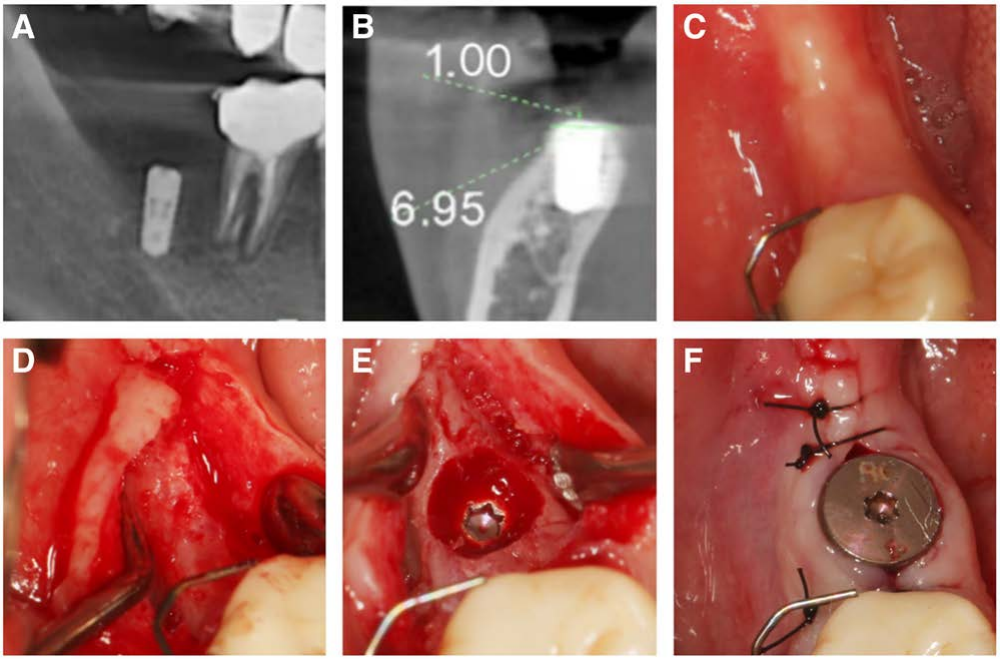
Reexamination 6 mo after operation. (A and B) The width of buccolingual bone 1 mm below the top of alveolar ridge is 6.95 mm. (C and D) After flipping the flap, the width of the buccal lingual side of the alveolar ridge is still acceptable. (E) Grind off 2 mm high bone and expose covering screws. (F) Install healing base and intermittently suture.

The patient visited our department 6 months after surgery for secondary implant repair. The right mandibular second molar mucosa healed well. CBCT images were taken, and the width of the buccal and lingual bone was measured to be 6.95 mm. Local anesthesia was performed at the top of the alveolar ridge, and the buccal and lingual mucoperiosteal flaps were opened to fully expose the bone surface of right mandibular second molar areas. The width of the buccal and lingual sides was acceptable, and no covering screws were seen. The bone at a height of 2 mm was removed, and the covering screws were exposed. The screwdriver removed the covering screws, rinsed with 0.9% normal saline, installed the ITI 6 mm × 6 mm healing abutment, sutured, 1 week after the return visit (Fig. 5).

## 3. Discussion

The patient complained of a 2-year history of anxiety, and regularly took fluvoxamine maleate tablets+quetiapine fumarate tablets+alprazolam tablets. Fluvoxamine maleate tablets are SSRIs, in addition to being used in patients with depression, it is also one of the commonly used drugs for patients with anxiety disorders.^[11]^ SSRIs exert pharmacological effects by inhibiting serotonin transporters to block serotonin reuptake and prolong extracellular activity.^[12]^ By studying the distribution of 5-hydroxytryptamine receptors and 5-hydroxytryptamine transporters in bones, the results showed that drugs that antagonize may affect bone metabolism, thereby forming drug-induced osteoporosis.^[13]^ An study evaluated the bone mineral density of the lumbar spine, total hip neck and femoral neck after taking SSRIs. The conclusion confirmed that SSRIs might affect bone metabolism and reduce bone mineral density.^[14]^ Another clinical trial on the relationship between SSRIs and fractures has shown that SSRIs are associated with an increased risk of fracture. Reduced bone mineral density observed in the SSRI experimental group may lead to changes in bone turnover and loss, affecting bone formation and absorption.^[15]^ Quetiapine fumarate tablets is a commonly used atypical antipsychotic drug, and quetiapine fumarate tablets can educes the occurrence of osteolytic bone loss induced by MDA-MB-231 cells by inhibiting osteoclast differentiation. Molecular analysis showed that it was by inhibiting the signaling pathway that quetiapine fumarate tablets inhibited osteoclast differentiation.^[16]^ Alprazolam is a short-acting drug with anti-anxiety, sedation, hypnosis, anti-convulsion and amnesia characteristics. Studies have shown alprazolam has little effect on osteogenesis and osteoclasts.^[17]^ The preoperative CBCT results showed that there was bone loss in the buccal side and alveolar crest of the right mandibular second molar, which was not excluded from the adverse reactions caused by long-term use of anti-anxiety drugs. Other studies have shown that anxiety disorder is related to the failure rate of implant implantation and postoperative wound recovery while affecting the mental health of patients.^[14]^ The causes may be related to the compliance of dental visits, poor oral hygiene and the use of anti-anxiety drugs. The relationship between mental health disorders, stress and peri-implant diseases is similar to that reported in periodontitis. Considering the interaction between pathogens and the host immune system, stress caused by psychosocial factors may affect periodontal biofilms.^[18]^ It has been reported that stress-related hormones may regulate bacterial growth and regulate the virulence factors of certain species, leading to the transformation to microecological disorders and affecting the health of periodontal tissues. The long-term curative effect, bone increment and soft tissue healing effect of oral implant restoration are closely related to the health of periodontal tissue.^[19]^ It is recommended that the safe distance between the bottom of the implant and the inferior alveolar nerve canal is ≥2.0 mm, and the buccal side of the implant has a alveolar bone thickness of ≥1.5 mm.^[20,21]^ When alveolar bone have defects, in the absence of bone graft technology, inclined, narrow or short implants may also be considered.^[22–24]^ Narrow-diameter implants have also been proved to be one of the solutions for two-way bone defects in the clinical application guidelines for International Team for Implantology (ITI),^[25]^ it shows that the mandibular second molar area bears a large chewing pressure, and the implant width is preferably ≥4.1 mm and the length is ≥8 mm.^[26]^ Otherwise, the implant is prone to fracture when the chewing function is exerted. However, in the case of bidirectional bone defects, the increment of the height and width of the alveolar ridge is necessary and can be achieved through a variety of surgical methods.^[11]^ These procedures include implanting bone grafts or stimulating bone formation in GBR. Autologous bone graft has good bone conductivity and osteoinductivity,^[27–30]^ and is considered to be the gold standard for reconstruction of bone defects.^[31]^ However, the donor site is limited, and trauma and complications are the biggest disadvantages it faces. For example, the bone mass of several donor sites is limited, additional surgery is required, and the incidence of complications at the donor site may increase, including pain, infection, loss of sensation and hematoma formation, and the increase in operation time and the unpredictability of postoperative bone resorption.

At present, various bone substitute materials have been put into clinical application, and these grafts are usually mixed with autologous bone.^[32]^ In clinical practice, microparticle xenografts are used to fill the gap of residual bone crest creating a stable regeneration environment. With the passage of time, it shows well bone stability.^[33–35]^ GBR with bone granule grafts is an alternative to reconstruct jaw defects. Bone substitute materials and collagen membranes are the most common materials in GBR. However, similar to the cases reported in this paper, it is difficult for collagen membranes to maintain a suitable and stable bone regeneration space under buccal muscle pressure in patients with alveolar crest and buccal bone defects. Because collagen membranes are easy to collapse and produce micro-motions that affect blood supply and bone graft materials are easy to move in the bone graft area to affect osteogenesis,^[36]^ which is one of the challenges we face. After more than 50 years of development, the clinical application of CGF as the latest generation of platelet concentrates has been recognized by many clinicians and CGF has been proposed as an auxiliary technology that can promote osteogenesis.^[37–39]^ Bone particles and GFs form a framework helps bone particles to be placed and stabilized in bone defects, and can be formed into the ideal shape at the bone defect.^[40]^

In 1997, Whitman et al^[41]^ published the first article on the application of the first generation of platelet-rich plasma in oral and maxillofacial surgery. In 2000, Choukroun et al^[42,43]^ discovered platelet rich fibrin, the injectability and ease-of-applicability of this biomaterial has led to its various clinical applications in the oral and maxillofacial bone regeneration such as ridge augmentation, sinus floor elevation, cleft palate reconstruction and so on. With the development of centrifugation technology, sacco reported the latest platelet concentrate CGF in 2006.^[43]^ CGF is an autologous platelet concentrate product. Some scholars believe that CGF provides a reliable biological scaffold and serves as a comprehensive reservoir for the slow release of tightly connected GFs, which helps to accelerate tissue regeneration.^[44]^ Compared with platelet-rich plasma and platelet rich fibrin, a denser and larger fibrin matrix can be isolated, and its GFs are richer. The dense 3-dimensional network of fibrin ensures the slow release of GFs. In addition, the increase in the number of CD34-positive cells in CGF indicates an improved ability to respond to neovascularization, collagen synthesis, epithelial and epidermal regeneration, and hemostatic response to injury. CGF can form a 3-dimensional fibrin network with bone conduction, providing a natural scaffold environment for local vascularization and new bone tissue growth.^[45]^ At the same time, a large number of autologous GFs are stably polymerized in the fibrin matrix, which can significantly increase the local sustained release of endogenous GFs.^[46]^ The release of GFs in CGF is stable and continuous, mimicking the physiological release process in the human body, so CGF is considered to be a natural growth factor carrier with good sustained release function.^[47]^ In recent years, some studies have combined tissue engineering materials with CGF, such as CGF combined with nano-hydroxyapatite, bone powder and mineralized collagen. The results show that CGF has a good effect on promoting osteogenesis.

A number of clinical trials have studied the effect of CGF on the repair of jaw defects. At different time points before and after surgery, serum bone alkaline phosphatase, osteocalcin and bone mineral density were measured, and regular examinations and computed tomography were performed during exploration or follow-up. The results showed that the prognosis of patients with CGF-assisted bone increment was better than that of conventional bone increment, as shown in Table 1.

**Table 1:** Clinical research reports of CGF combined with GBR technique.

Author and year	Included number of people	Research object	Follow-up	Healing evaluation method	Result
Atef M et al^[48]^ 2022	Experimental group: 14 cases Control group: 14 cases	Experimental group: CGF and GBR Control group: GBR	6 mo	CBCT	The average bone width was 9 ± 0.71 mm in the experimental group and 7.9 ± 0.92 mm in the control group after 6 mo. The primary stability of the experimental group is high
Lin Yong et al^[49]^ 2020	Experimental group: 42 cases Control group: 41 cases	Experimental group: CGF and Bio-Oss bone powder Control group: Bio-Oss bone meal and blood	7 d, 6 wk, 1 yr	Probe, X-ray film	The bleeding index, probing depth, bone graft height and osteogenic height of the experimental group were significantly better than those of the control group (*P* < .05)
Aizawa Het al.^[50]^ 2020	Experimental group: 20 cases Control group: 20 cases	Experimental group: CGF and Bio-Oss bone powder Control group: Bio-Oss bone powder	1 wk, 12 wk, 5 mo	Serum BAP, osteocalcin and bone mineral density were measured. Computer tomography scanning	The levels of BAP and osteocalcin in the 2 groups were significantly higher than those in the control group at 1 and 12 wk after operation (all *P* < .05). The bone mineral density in the bone defect area of the experimental group was also significantly higher than that of the control group at 5 mo after operation (*P* < .05)
Wang et al^[51]^ 2020	Experimental group: 20 cases Control group: 20 cases	Experimental group: CGF+bone powder Control group: bone powder+collagen membrane	6 mo	CBCT	The increase of bone width in the observation group was 3.70 ± 0.28 mm, and that in the control group was 2.96 ± 0.16 mm. The bone increment in the observation group was greater than that in the control group, and the difference was statistically significant (*P* = .000).

BAP = bone alkaline phosphatase, CGF = concentrated growth factor, GBR = guided bone regeneration.

CGF plays an important role in restoring stable bone mass around the implant, in addition to reducing swelling and pain in the surgical area, thereby improving comfort. Studies have found that CGF can not only guide bone regeneration, but also help to reduce postoperative edema and peri-implantitis recurrence.^[52]^ A retrospective study by Dai et al^[53,54]^ showed that the combined application of GBR and CGF not only made the effect of bone regeneration preferably, but also greatly shortened the soft tissue healing time, and showed satisfactory results in alleviating postoperative discomfort. Yu et al^[54]^ observed that CGF significantly reduced postoperative swelling in patients with single maxillary anterior teeth loss, but had no significant effect on pain relief. Recently, Taschieri et al^[55,56]^ conducted a prospective comparative study to compare the application of implant alone and implant combined with CGF, in order to determine the effect of CGF on quality of life. Consistent with our observation results and the observation results of most experts in oral implant repair field, the quality of life of patients treated with CGF was significantly higher during the recovery period after implant implantation. Since the discovery of CGF, most studies have shown that they can effectively promote bone regeneration. The focus of the study is to use these bioactive molecules to induce new bone formation. The US Food and Drug Administration has approved the use of CGF in dentistry. Different GFs in different clinical and experimental conditions will show the ability to induce bone formation by stimulating the osteogenic differentiation of MSCs, enhancing osteoblast proliferation and extracellular matrix biosynthesis.^[57,58]^ Therefore, these evidence that CGF plays an important role in bone regeneration.

## 4. Conclusion and prospects

The application of CGF combined with GBR technique for dental implant surgery in patients with anxiety disorders of dentition defect at the implant simultaneous bone increment, so that anti-anxiety drugs did not significantly affect the treatment effect of bone increment. The study showed that CGF had an ideal osteogenic effect. While the patient continued to take anti-anxiety drugs, we used CGF combined with GBR technology to achieve the ideal osteogenic effect in the simultaneous alveolar bone increment of mandibular molar implantation. CGF combined with GBR technology has played a positive role in the bone increment of such special patients. It may be an ideal method for implant restoration and alveolar bone increment in patients with dentition defect and taking anti-anxiety drugs. However, there are some limitations in this study, the sample size is small, the observation time is relatively short, and more clinical trials are still needed to determine its efficacy.

## Author contributions

**Conceptualization:** Wen Luo, Chao Yang.

**Funding acquisition:** Wen Luo.

**Investigation:** Wen Luo, Zhixin Li, Jinrong Wang, Kaiyue Zheng.

**Methodology:** Wen Luo.

**Project administration:** Wen Luo.

**Writing – review & editing:** Wen Luo, Chao Yang.
